# A phenomenological base semi-physical thermodynamic model for the cylinder and exhaust manifold of a natural gas 2-megawatt four-stroke internal combustion engine

**DOI:** 10.1016/j.heliyon.2019.e02700

**Published:** 2019-10-22

**Authors:** Guillermo Valencia Ochoa, Cesar Isaza-Roldan, Jorge Duarte Forero

**Affiliations:** a Programa De Ingeniería Mecánica, Universidad Del Atlántico, Carrera 30 Número 8-49, Puerto Colombia, Barranquilla, 080007, Colombia; b Programa de Ingeniería Mecánica, Universidad Pontificia Bolivariana, Medellín, 050004, Colombia

**Keywords:** Applied mathematics, Mechanical engineering, Thermodynamics, Energy conservation, Mathematical modeling, Mean value model, Natural gas, Phenomenological base semi-physical model, Power generation, Spark ignition engine

## Abstract

This paper presents the application of a systematic methodology to obtain a semi-physical model of phenomenological base for a 2 MW internal combustion engine to generate electric power operating with natural gas, as a function of the average thermodynamic value normally measured in industrial applications. Specifically, the application of the methodology is focused on the cylinders, exhaust manifold, and turbocharger turbine sections. The proposed model was validated with actual operating data, obtaining an error rate not exceeding 5%, which allow a thermal characterization of the Jenbacher JMS 612 GS-N based on the model. A parametric analysis is conducted; considering the volumetric efficiency, the output electric power, the effective efficiency, the exhaust gas temperature, the turbine mass flow, the specific fuel consumption under the nominal operation conditions, which is 1.16 bar in the gas pressure, 65 °C in the cooling water temperature, 35 °C in the average ambient temperature, and 1500 rpm. The results of this model can be used to evaluate the thermodynamic performance parameters of waste heat recovery systems. On the other hand, new control strategies and the implementation of state observers for the detection and diagnosis of failures can be developed based on the proposed model.

## 1 Introduction

The internal combustion engines (ICE) modeling is mainly based on thermodynamic models used to predict the properties and variables of interest that generally are difficult to measure [1]. The importance of the different models used for the systems and equipment simulation dependent on thermodynamic variables lies in the fact of the continuous development of improvements for them, such as the efficient and rational use of energy [2]. In the ICE case, many authors have developed models to describe the engines phenomenology. To understand more about the ICE modeling, it is necessary to know that there are different types of model families, which differ in the way of describing and affecting the treatment of the fuel energy contribution, specifically in the behavior of the rate of heat release. To simplify the classification of these models, at the level of those that are zero-dimensional, they are generally categorized into two types, cylinder by cylinder models and mean value models [3]. The first ones have a detailed description of all the processes that occur within the cylinder throughout the cycle, such as combustion or fuel injection, also can even be classified into two types, predictive models [4, 5] and those of diagnostic [1, 6]. Being the first dependents of the latter, since, in the diagnostic models, the engine heat release rate is obtained [1], which is the main input of the predictive models [4]. These types of models are usually used for the evaluation, design, and development of ICE, due to the high degree of detail considered in all the strokes, such as the description of the heat transfer release [7, 8] and [9], engine models of a varying range of accuracy and computational time can be employed depending on the required application [10, 11]. Concerning to the mean value engine model (MVEM), it is not necessary and is not desired a dependence of the thermodynamic variables associated to the four strokes presented in a normal power generation cycle of an ICE. Since is only desired average values or values from mass and energy balances of simplified engine sub-systems [12, 13], this to facilitate future engine control and observation applications [14, 15] in real time. In the development of the mathematical description of the engine, more opts for MVEM [23, 24, 25, 26, 27, 28, 29], this type of modeling does not present high complexity compared to those of cylinder per cylinder, however, allow to obtain results of the macroscopic behavior of the engine, and its component [3, 12, 16] and [17]. Hendricks, in 1990 [12], developed an MVEM in an ICE by provoked ignition, with energy balances based on classical thermodynamics, very similar to what is normally implemented today. Likewise, Hendricks, in view of the fact that the first attempts of this type of models [18, 19] did not present a varied applicability, and that in addition his previous works were for two-stroke diesel engines [20, 21], developed a work with a detailed explanation of the semi-physical model, capable of being implemented with data obtained from a previous modeling, or from experimental data taken from the different points of operation of the engine.

Based on the bibliographic review, it is shown that several authors have contributed to the MVEM of ICE, however, there are no records of the application of the phenomenological-based semi-physical modeling methodology (PBSM) [22] to the modeling of stationary generation engines, which allow studying the behavior of each of the subsystems of the process, in addition to evaluating the energy availability of exhaust gases. As examples, there are works applied to basic cases of chemical processes such as fermentation [23], evaporation [22], among others [24], but in the literature available there are not register of the application of this methodology to obtain mean value engine model of a natural gas engine.

S. Soylu et al. [25] used a two-zone thermodynamic model with zero dimensions to determine the operating conditions restricted by the cylinders of an ICE. This work is related by the type of area covered by the research; in addition, it shows a model that offers the possibility to select the operational parameters according to the working conditions which provides a crucial tool in the design of natural gas engines. Another related work was developed by U. Sohail et al. [26] studied the combustion process in a computerized premix form to analyze the influence in the variations of pressure drop and other significant parameters for the model. This last author did not analyze the influence of natural gas concentration on the combustion efficiency which suggests that he failed to consider in detail the properties of the working fluid. However, it makes a significant contribution to education. In addition, G. Valencia et al. [27] simulated a semi-physical model by means of fundamental equations to predict the thermal behavior of the engine under study, and the dynamics of the airline of the 2 MW Jenbacher natural gas internal combustion engine is presented, but focused on the engine intake area only, which indicates a limitation of the models with respect to the system parameters that were not analyzed.

The main contribution of this paper is to present the development of a detail PBSM of a natural gas 2 MW four-stroke internal combustion engine widely used in the industrial sector. The steps to obtain the dynamic model is presented, and the validation was conducted with experimental data. The evaluation of internal variables and the main output of the model have been studied in a typical operation day of the engine.

## 2 Methodology

### 2.1 Phenomenological base semi-physical methodology

**Fig. 1:**
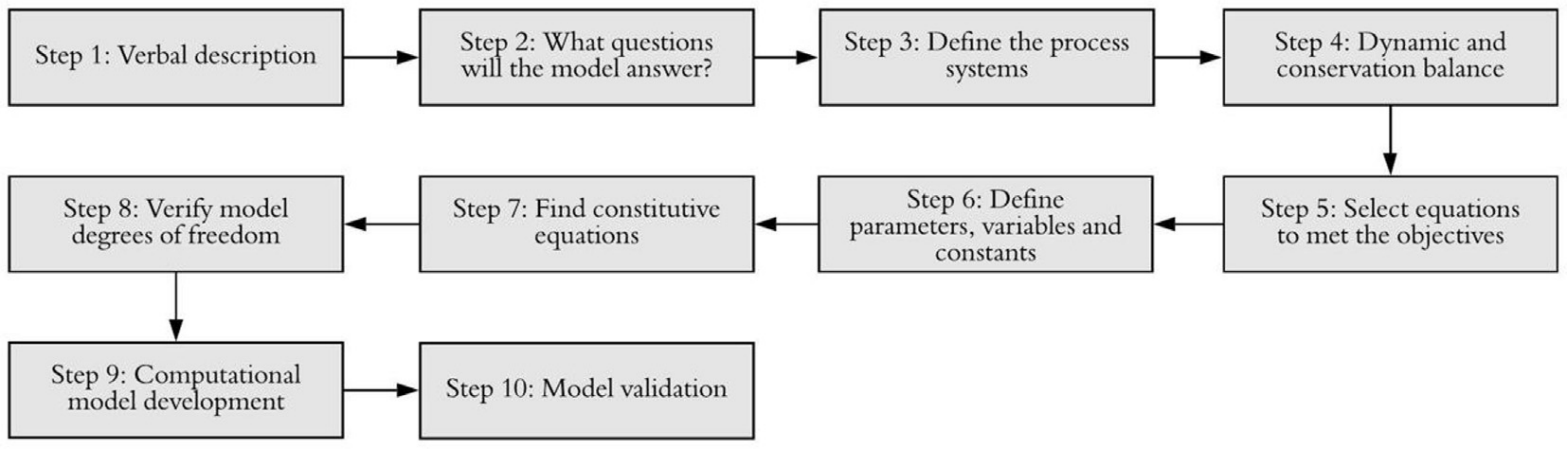
Methodology used in this research.

In this work, the methodology for obtaining a PBSM [22, 24, 28] is applied to perform a quick and correct analysis of different representative variables of the engine performance in real time with a potential to be implemented in control strategies. This methodology consists of 10 steps, as shown in Fig. 1 [22], which are described as follows. Step 1: Describe verbally and using a flow chart of processes that complement each other. Step 2: Set a detail level for the model, according to its use: What questions will the model answer? Step 3: Define as many process systems (PS) within the process to be modeled, as required by the detail level, and represent the relationship of all subsystems of the process in a block diagram. Step 4: Apply the principle of conservation of mass and energy to each process system. The set of equations obtained in this step are called “Dynamic Balance Equations” (DBEs) Step 5: Select from the DBEs those with valuable information to meet the objective of the model. Step 6: Define, for the essential DBEs, the parameters, variables, and known constants of each PS. Step 7: Find constitutive equations that allow the calculation of the most significant parameters in each process system. Step 8: Verify the model degrees of freedom. Step 9: Obtain the computational model or solution of the mathematical model. Step 10: Validate the model for different conditions and evaluate its performance.

### 2.2 Engine and fuel characteristics

**Fig. 2:**
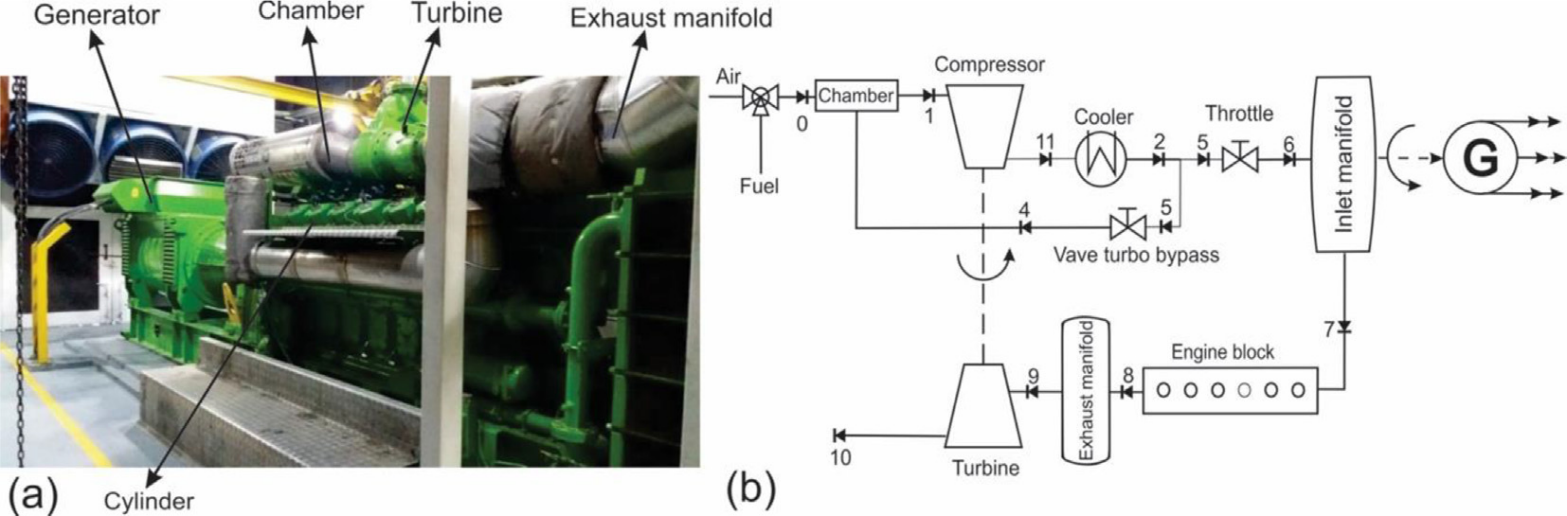
Engine-Generator, a) Parts and section distribution, b) Engine-Generator illustration used for model development.

The 2 MW Jenbacher JMS 612 GS-N.L natural gas engine studied in this research is shown in Fig. 2a, which is widely used for autogeneration purposes worldwide [29], given its versatility in application to petroleum, textile, cement, pharmaceuticals, plastics, and paper industries.

The selected application for the study is an engine installed in a company in the plastic sector in the city of Barranquilla - Colombia without any waste heat recovery system to improve efficiency. The engine regulates fuel consumption to operate between a minimum load of 1000 kWe and a maximum load of 1982 kWe, with an air/fuel ratio ranging from 1.79 to 1.97. The engine load is changed recirculating the air-gas mixture flow through the turbo bypass valve, as shown in Fig. 2b. Exhaust gases (state 8) are generated in each of its 12 cylinders with a temperature ranging between 580 °C and 650 °C, which at the outlet of the turbine are between 420 °C and 460 °C.

The composition of the fuel used where the plastic plant operates is mainly 97.97% CH_4_, 1.5% N_2_, 0.25% C_2_H_6_ and 0.16% CO_2_, whose mixture with the air is given at a line pressure between 1.152 bar to 1.211 bar, and uncorrected volumetric ratio of 110 L/s to 140 L/s to obtain an optimal flammable gas-air mixture.

## 3 Model

Although the focus of this work is associated with the cylinder and engine exhaust sections, the first three steps of the methodology are identical to a model generated for the mathematical and thermodynamic representation of the intake section of the same engine. Therefore the first two steps and part of the third of the previously chosen methodology [27] and the general information of the engine, will be referenced from previous work [27]. The natural gas composition in this location is 97.97% CH_4_, 1,5% N_2_, 0.1626% CO_2_, 0.2559% C_2_H_6_, 0.0516% C_3_H_8_, 0.0207% C_4_H_10_ (i-butane), 0.0083% C_4_H_10_ (n-butane), 0.0076% C_5_H_12_ (i-pentane), 0.0021% C_5_H_12_ (n-pentane) and 0.0132% C_6_H_14_ (n-hexane) with a pressure line comprised between 1152 bar at 1211 bar. The steps application of the methodology is presented as follow.

### 3.1 Questions the model will answer

Taking into account the previous relevant information [27], the questions that the model will answer will be the following ones:

• ¿How does heat transfer affect the effective efficiency and the mean effective pressure of the engine?
• ¿How does the cylinder outlet temperature, and the inlet temperature of the compressor of the turbocharger influence the outlet temperature of the turbine?

### 3.2 Apply the principle of mass and energy conservation to each process system

Once the model objective is defined, the mass and energy conservation principles are applied to each subsystem of interest [27], according to the following considerations:

• The intake and exhaust manifolds are considered as reservoirs of thermal energy, with output properties equal to those of the control volume.
• The two systems of turbochargers are unified in a single system, which will be modeled as a single stage of expansion in the exhaust.
• One cylinder is studied, and it is assumed that its behavior is the same for the other cylinders, where the exhaust gas output properties will be the same as those contained within it.

With the above, it is necessary to know the amount of net mass that enters and leaves the engine cylinders and their respective thermodynamic properties associated with first-law thermodynamic balances. For this reason, it is extremely important to know three variables, which are the fuel mass flow, the air mass flow and the flows temperatures present in the systems to be analyzed, given that those variables have a significant impact on the overall engine performance [27, 30].

#### 3.2.1 Turbocharger

For the mass balance in the compressor, just one mass flow enters ${\dot{m}}_{1}\left(t\right)$ and a single mass flow leaves${\overset{˙}{\;m}}_{2}\left(t\right)$, therefore the mass dynamics in the compressor $m_{com}\left(t\right)$ is calculated with Eq. (1) as

(1) $$\frac{dm_{com}\left(t\right)}{dt}={\dot{m}}_{1}\left(t\right)-{\dot{m}}_{2}\left(t\right)$$

Regards to the energy balance in this equipment, the energy consumed by the compressor ${\dot{W}}_{com}\left(t\right)$ is described as a function of the difference in energy of the fluid as shown in Eq. (2) [31].

(2) $${\dot{W}}_{comp}\left(t\right)=\frac{{\dot{m}}_{1}\left(t\right)}{\eta_{comp}}\frac{k\cdot R_{1}\left(t\right)\left(T_{comp}\left(t\right)-T_{1}\left(t\right)\right)}{k-1}$$

For the dynamics of the turbine mass$m_{turb}\left(t\right)$, a similar analysis to the compressor was performed as shown in Eq. (3), where ${\dot{m}}_{9}\left(t\right)$ is the exhaust mass flow of the exhaust manifold and is the exhaust gas mass flow delivery to the environment [31].

(3) $$\frac{dm_{turb}\left(t\right)}{dt}={\dot{m}}_{9}\left(t\right)-{\dot{m}}_{10}\left(t\right)$$

The energy balance of the turbine allows determining the energy produced ${\dot{W}}_{turb}\left(t\right)$, which is calculated by Eq. (4) as.

(4) $${\dot{W}}_{turb}\left(t\right)={\dot{m}}_{9}\left(t\right)\cdot C_{ptur}\left(t\right)\cdot T_{9}\left(t\right)-{\dot{m}}_{9}\left(t\right)\cdot C_{ptur}\left(t\right)\cdot T_{10}\left(t\right)$$

#### 3.2.2 Cylinder

For the gases contained within the engine cylinders, basic balances of first-law energy and mass are applied to the control volume. For the cylinder mass balance only are considered two flows, one incoming ${\dot{m}}_{7}\left(t\right)$ and another outgoing ${\dot{m}}_{8}\left(t\right)$ resulting in Eq. (5) as

(5) $$\frac{dm_{cyl}\left(t\right)}{dt}={\dot{m}}_{7}\left(t\right)-{\dot{m}}_{8}\left(t\right)$$

The energy balance applied to the cylinder as shown in Eq. (6) allow to determinate the exhaust gas temperature dynamic $T_{8}\left(t\right)$, the control volume input and output enthalpies, the outgoing effective work, the energy losses of the gases to the walls by heat transfer, the energy generated due to fuel ignition and the change in the internal energy of the gases contained in the cylinder are taken into account:

(6) $$m_{cyl}\left(t\right)\cdot {C_{v}}_{8}\left(t\right)\frac{dT_{8}\left(t\right)}{dt}={\dot{Q}}_{in}\left(t\right)-{\dot{Q}}_{loss}\left(t\right)-{\dot{W}}_{out}\left(t\right)+{\dot{m}}_{7}\left(t\right)\cdot R_{7}\left(t\right)\cdot T_{7}\left(t\right)-{\dot{m}}_{8}\left(t\right)\cdot R_{8}\left(t\right)\cdot T_{8}\left(t\right)$$

With these approaches, the energy and mass balances of the subsystems in question are completed. Some variables are shown in the equations previously were not presented in this step because they are obtained from constitutive expressions that do not correspond to balances. Therefore, they will be shown in future steps according to the proposed methodology.

### 3.3 Select from the DBEs those with valuable information to meet the objective of the model

#### 3.3.1 Turbocharger

From Eqs. (1), (2), (3), and (4) are the mass balances of the compressor and the turbine. These elements are not considered as mass reservoirs, considering that those elements do not accumulate mass. Therefore, the equations above remain in Eqs. (7) and (8) as

(7) $${\dot{m}}_{2}\left(t\right)={\dot{m}}_{1}\left(t\right)$$

(8) $${\dot{m}}_{10}\left(t\right)={\dot{m}}_{9}\left(t\right)$$

Since the model consider a total energy transfer from the turbine to the compressor, it is necessary to consider Eqs. (9) and (10) as

(9) $${\dot{W}}_{com}={\dot{W}}_{turb}$$

(10) $$\frac{{\dot{m}}_{1}\left(t\right)}{\eta_{comp}}\left(\frac{k\cdot R_{1}(t)\cdot \left(T_{comp}\left(t\right)-T_{1}\left(t\right)\right)}{k-1}\right)={\dot{m}}_{9}\left(t\right)\cdot C_{ptur}\left(t\right)\cdot T_{9}\left(t\right)-{\dot{m}}_{9}\left(t\right)\cdot C_{ptur}\left(t\right)\cdot T_{10}\left(t\right)$$

#### 3.3.2 Cylinder

From Eqs. (5) and (6), only (5) represents the mass balance of the gas inside the cylinder. Because of a macroscopic balance is made in a measurable time scale, the mass rate change inside of the cylinders remains almost constant as a consequence of the engine constant regime operation. Therefore Eq. (5) results as shown in Eq. (11) as follow

(11) $${\dot{m}}_{7}\left(t\right)={\dot{m}}_{8}\left(t\right)$$

Regarding the energy balance of this system, the model proposed in step 4 of the methodology does not receive an additional simplification, therefore, it can be deduced that Eqs. (7), (8), (10), and (11) are the equations that allow explaining the information of interest of this subsystem.

### 3.4 Define, for the essential DBEs the parameters, variables and known constants of each PS

The variables for the essential DBEs are the mass flows of each process system ${\dot{m}}_{1}\left(t\right),{\overset{˙}{\;m}}_{2}\left(t\right)$, ${\dot{m}}_{9}\left(t\right)\;$and ${\dot{m}}_{10}\left(t\right)\;$for the turbocharger, ${\dot{m}}_{7}\left(t\right)\;$and ${\dot{m}}_{8}\left(t\right)\;$for the cylinder. In addition, energy flows associated with different thermodynamic phenomena are essential, such as the temperatures, output work, and energy transfer by heat. In this sense, $T_{1}\left(t\right)$, $T_{2}\left(t\right),\text{ ​ ​}T_{9}\left(t\right)$, $T_{10}\left(t\right)$, ${\dot{Q}}_{per}\left(t\right)$, ${\dot{W}}_{com}$ and ${\dot{W}}_{turb}$ are essential variables. As an essential parameter from the equations presented, the output work ${\dot{W}}_{out}\left(t\right)\;$and the input heat ${\dot{Q}}_{in}\left(t\right)$ are considered, since from these parameters many of the variables of interest of this work can be calculated. Related to the known constants of each PS only the thermodynamic properties of the flows were defined, such as the specific heats and their ideal gas constants.

### 3.5 Find constitutive equations that allow the calculation of the most significant parameters in each process system

Considering the framework of these paper, which focuses on developing a phenomenological semi-physical mean value engine model specifically in the cylinder and exhaust gas subsystems, additional equations are required to those raised in the steps 1 to 6 of the methodology. To obtain a correct implementation of the model, each one of these following equations will be considered according to their respective subsystem.

#### 3.5.1 Turbocharger

For this subsystem, an equation that relates the temperatures and pressures of the compressor are needed, for this process an isentropic state change formulation is proposed, taking into account their respective efficiency, as shown below in Eq. (12) [31].

(12) $$\frac{T_{comp}\left(t\right)}{T_{1}\left(t\right)}=1+\frac{1}{\eta_{comp}}\left({\left(\frac{P_{2}\left(t\right)}{P_{1}\left(t\right)}\right)}^{\frac{k-1}{k}}-1\right)$$

#### 3.5.2 Cylinder

This subsystem has not the capacity to accumulate mass. The dynamics associated with the pressure and temperature inside it is a consequence of the fundamental fuel combustion process carried out in an internal combustion engine. Due to the presence of this phenomenon of this subsystem, it is necessary to denote the effect it has on the usually studied variables, more specifically the temperature. As a first part, it is necessary to calculate the flows associated with the cylinder according to Eqs. (13) and (14) as

(13) $${\dot{m}}_{7}\left(t\right)={\dot{m}}_{air}\left(t\right)+{\dot{m}}_{fuel}\left(t\right)$$

(14) $${\dot{m}}_{air}\left(t\right)={\dot{m}}_{fuel}\left(t\right)\cdot AFR\cdot \lambda$$

Once the mass flows are obtained, the energy balance of the gas according to the first thermodynamic law is applied to obtain the rate of change of temperature at the exit of the cylinder as shown in Eq. (15), taking into account the combustion phenomenon of the gas inside the cylinder

(15) $$\frac{dT_{8}\left(t\right)}{dt}=\frac{{\dot{Q}}_{in}\left(t\right)-{\dot{Q}}_{loss}\left(t\right)-{\dot{W}}_{out}\left(t\right)+{\dot{m}}_{7}\left(t\right)\cdot R_{7}\left(t\right)\cdot T_{7}\left(t\right)-{\dot{m}}_{8}\left(t\right)\cdot R_{8}\left(t\right)\cdot T_{8}\left(t\right)}{m_{cil}\left(t\right)\cdot {C_{v}}_{8}\left(t\right)}$$

where ${\dot{Q}}_{in}\left(t\right)$, is the heat of combustion generated by the fuel ignition, the heat rejected due to the convective heat transfer phenomenon between the gases inside the combustion chamber and the cylinder walls and the engine effective output energy respectively. The fourth and fifth terms of the numerator in Eq. (15) corresponds to the enthalpy generated by the incoming and outgoing mass flows of the cylinder. Eq. (16) is used to calculate the inlet mass to the cylinder [30].

(16) $$m_{cyl}\left(t\right)=\eta_{v}\left(t\right)\cdot V_{d}\cdot \rho_{7}\left(t\right)$$

where $\eta_{v}\left(t\right)$ represents the volumetric efficiency, which is obtained from Eq. (17) as

(17) $$\eta_{v}\left(t\right)=0.24354-0.001371\cdot P_{7}\left(t\right)+0.00035484\cdot T_{7}\left(t\right)+0.00013084\cdot \omega_{e}\left(t\right)+5.1829\cdot {\dot{V}}_{fuel}\left(t\right)$$

For ${\dot{Q}}_{in}\left(t\right)$ is used Eq. (18) as.

(18) $${\dot{Q}}_{in}\left(t\right)=m_{fuel}\left(t\right)\cdot LHV\cdot \frac{RPM}{120}\text{,}$$

where $m_{fuel}\left(t\right)$ and LHV is the amount of fuel mass and its calorific value respectively, and RPM are the engine revolutions per minute. Regarding ${\dot{Q}}_{loss}\left(t\right)$ as shown in Eq. (19), linear regression is proposed that takes into account the variables ${\dot{W}}_{comp}\left(t\right)\;$and ${\dot{V}}_{fuel}\left(t\right)$, which are the electric energy delivered by the generator and the gas volumetric flow, since at the moment of the heat transfer process characterization of through what was developed by Woschni [8, 9] it was found that these variables had a considerable weight in the behavior of the heat rejected rate of the engine.

(19) $${\dot{Q}}_{loss}\left(t\right)=\beta_{1}\cdot {\dot{V}}_{fuel}\left(t\right)+\beta_{2}\cdot {\left({\dot{V}}_{fuel}\left(t\right)\right)}^{2}+\beta_{3}\cdot {\left({\dot{V}}_{fuel}\left(t\right)\right)}^{3}+\beta_{4}\cdot {\overset{˙}{W}}_{out}\left(t\right)+\beta_{5}\cdot {\dot{W}}_{out}\left(t\right)\cdot {\overset{˙}{V}}_{fuel}\left(t\right)+\beta_{6}\cdot \frac{{\dot{W}}_{out}\left(t\right)}{{\dot{V}}_{fuel}\left(t\right)}+\beta_{7}\cdot {\overset{˙}{W}}_{out}\left(t\right)\cdot {\left({\dot{V}}_{fuel}\left(t\right)\right)}^{2}+\beta_{8}\cdot {\overset{˙}{W}}_{out}\left(t\right)\cdot {\left({\dot{V}}_{fuel}\left(t\right)\right)}^{3}+\beta_{9}\cdot {\overset{˙}{Q}}_{in}\left(t\right)\text{ ​}+\beta_{10}\cdot {\left({\dot{Q}}_{in}\left(t\right)\right)}^{2}+\beta_{11}\cdot {\dot{V}}_{fuel}\left(t\right)\cdot {\overset{˙}{Q}}_{in}\left(t\right)+\beta_{12}\cdot \frac{{\dot{Q}}_{in}\left(t\right)}{{\dot{W}}_{out}\left(t\right)}+\beta_{13}\cdot {\left({\dot{Q}}_{in}\left(t\right)\right)}^{3},$$

The regression coefficients of Eq. (19) are presented in Table 1. To obtain these values was needed a statistical analysis based on linear regression estimation, from this analysis could be concluded with a 95% confidence, that Eq. (19) predicts a 99% of the 2752 observations used. In this paper is only needed an equation that describes the heat transfer losses phenomena, due to this, a more detailed statistical analysis will be present in further works.

**Table 1:** Regression coefficients for estimating the heat transfer rate.

Coefficient	Value	Coefficient	Value
$\beta_{1}$	-1.19 × 10^2^	$\beta_{8}$	6.38 × 10^−6^
$\beta_{2}$	1.74	$\beta_{9}$	-2.96 × 10^1^
$\beta_{3}$	-6.77 × 10^−3^	$\beta_{10}$	4.11 × 10^−2^
$\beta_{4}$	2.06 × 10^2^	$\beta_{11}$	4.12 × 10^−2^
$\beta_{5}$	-2.31	$\beta_{12}$	1.23 × 10^3^
$\beta_{6}$	-3.37 × 10^3^	$\beta_{13}$	-3.23 × 10^−5^
$\beta_{7}$	9.14 × 10^−3^		

The mechanical work ${\dot{W}}_{out}\left(t\right)$ is estimated from Eq. (20) as a function of the generator efficiency $\eta_{e}$ and the electrical work ${\dot{W}}_{elec}\left(t\right)$

(20) $${\dot{W}}_{out}\left(t\right)=\frac{{\dot{W}}_{elec}\left(t\right)}{\eta_{e}}\;$$

To measure the performance of the engine, there are many variables that can be used for this purpose, but the particular case of this work will be addressed only the engine effective efficiency and its volumetric efficiency as showed in Eq. (17). The effective engine efficiency is calculated according to Eq. (21) [32] as

(21) $$\eta_{e}=\frac{{\dot{W}}_{out}\left(t\right)}{{\dot{Q}}_{in}\left(t\right)}\;$$

Replacing Eq. (18) and Eq. (20) in Eq. (21) is obtained Eq. (22) as

(22) $$\eta_{e}=\frac{{\dot{W}}_{elec}\left(t\right)}{\eta_{elec}\cdot m_{fuel}\left(t\right)\cdot LHV\cdot \frac{RPM}{60}}\;$$

The specific fuel consumption of the engine (BSFC), is another variable that allows to measure the performance of the engine and is calculated using Eq. (23) as

(23) $$BSFC\left(t\right)=\frac{{\dot{m}}_{fuel}\left(t\right)\cdot 1000\cdot 3600}{{\dot{W}}_{out}\left(t\right)}\;$$

#### 3.5.3 Exhaust manifold

For the exhaust manifold subsystem, a thermal resistance model was proposed to calculate the heat loss and the exhaust manifold outlet temperature from the Eqs. (24, 25, 26, 27, 28, 29, 30, 31, 32, 33, 34)

(24) $$h_{exh}\left(t\right)=21{(\vartheta_{8}\left(t\right))}^{0.52},$$

(25) $$h_{amb}\left(t\right)=28.6\text{ ​},$$

(26) $${\dot{m}}_{9}\left(t\right)={\dot{m}}_{8}\left(t\right)\cdot n_{cyl}\;,$$

(27) $$R_{exh}\left(t\right)=\frac{e_{exh}}{k_{exh}\cdot A_{exh}}\;,$$

(28) $$R_{ais}\left(t\right)=\frac{e_{ais}}{k_{ais}\cdot A_{ais}}\;,$$

(29) $$R_{exhconv}\left(t\right)=\frac{1}{h_{exh}\left(t\right)\cdot A_{exh}},$$

(30) $$R_{ambconv}\left(t\right)=\frac{1}{h_{exh}\left(t\right)\cdot A_{ais}}\;,$$

(31) $$R_{tot}\left(t\right)=R_{exh}\left(t\right)+R_{ais}\left(t\right)+R_{exhconv}\left(t\right)+R_{ambconv}\left(t\right)\;\text{,}$$

(32) $${\dot{Q}}_{perexh}\left(t\right)=\frac{T_{exhprom}\left(t\right)-T_{amb}\left(t\right)}{R_{tot}\left(t\right)}\;,$$

(33) $$m_{esc}\left(t\right)=\rho_{8}\left(t\right)\cdot \forall_{esc}\;,$$

(34) $$\frac{dT_{9}\left(t\right)}{dt}=\frac{{\dot{m}}_{9}\left(t\right)\cdot C_{ppromesc}\left(t\right)\cdot T_{8}\left(t\right)-{\dot{m}}_{9}\left(t\right)\cdot C_{ppromesc}\left(t\right)\cdot T_{9}\left(t\right)-{\dot{Q}}_{perexh}\left(t\right)}{m_{esc}\left(t\right)\cdot C_{vpromesc}\left(t\right)}\;\text{,}$$

where $h_{exh}\left(t\right)$ and $h_{amb}\left(t\right)$ are the convection heat transfer coefficients by the gases within the exhaust manifold and the surrounding air, respectively. $R_{exh}\left(t\right)$, $R_{ais}\left(t\right)$, $R_{exhconv}\left(t\right)$ and $R_{ambconv}\left(t\right)$ are the thermal resistances of the exhaust manifold, the heat insulator, the gases within the exhaust manifold and the surrounding air. ${\dot{Q}}_{perexh}\left(t\right)$ is the mean heat transfer rate of the exhaust manifold to the surroundings, $T_{exhprom}\left(t\right)$ is the average temperature of the exhaust gases within the exhaust manifold, $e_{exh}$ and $e_{ais}$ are the thicknesses of the exhaust manifold and the heat insulator, $k_{exh}$ and $k_{ais}$ are the heat conductivity of the elements mentioned above and therefore $A_{exh}$ and $A_{ais}$ are the respective heat transfer areas, $\forall_{esc}$ is the exhaust manifold volume, ${\dot{m}}_{9}\left(t\right)$ is the inlet and outlet mass flow. Finally, $T_{8}\left(t\right)$ and $T_{9}\left(t\right)$ are the gas temperatures at the inlet and outlet exhaust manifold.

### 3.6 Verify the model degrees of freedom

**Fig. 3:**
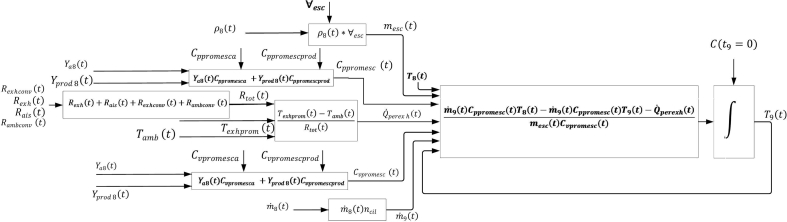
Exhaust manifold model representation.

In this step is verified that the number of equations coincides with the number of unknown variables of the model, also, relating the total number of unknowns raised an object of study. Fig. 3 shows the information flow chart representing the dynamic model of the engine exhaust manifold. Arrows starting in black dots symbolize parameters or constants, while normal arrows indicate model variables and parameters.

### 3.7 Obtain the computational model or solution of the mathematical model

For the calculation of the simultaneous differential and algebraic equations shown above, Matlab R2018b® was used, using the "ODE" commands, which in turn are based on high-order Runge-Kutta methods.

### 3.8 Validate the model for different conditions and evaluate its performance

**Fig. 4:**
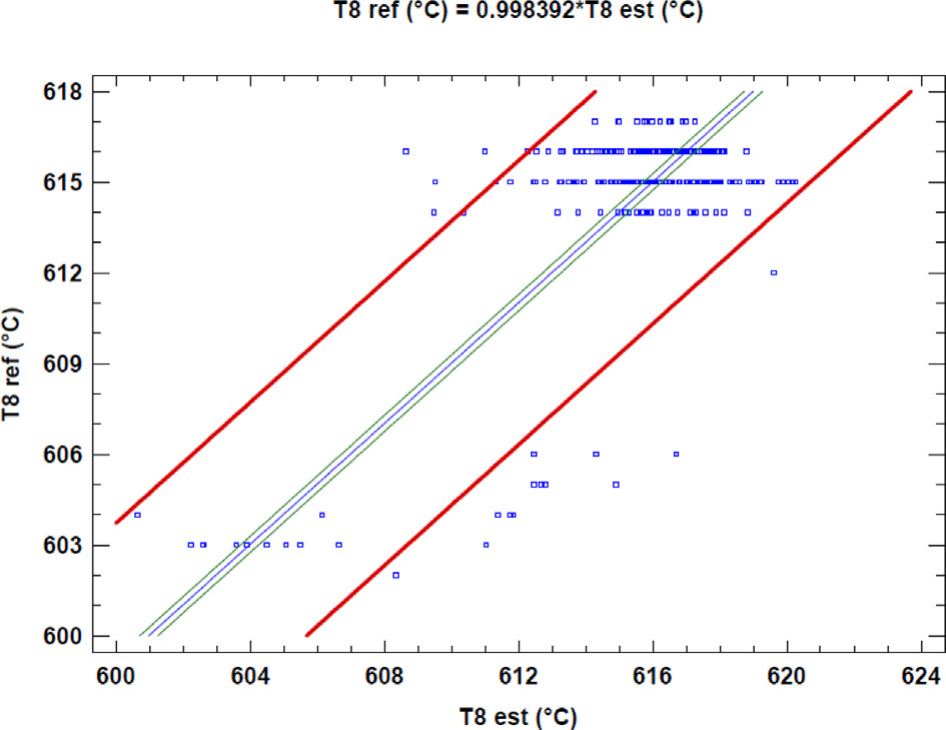
Linear regression between T8 reference and T8 estimated data.

**Fig. 5:**
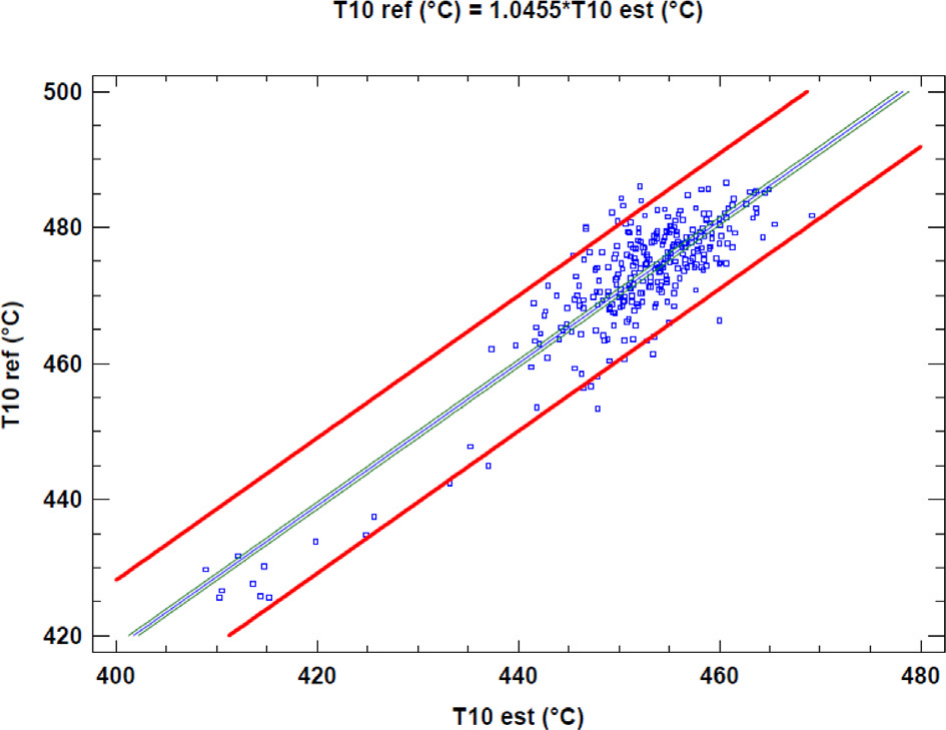
Linear regression between T10 reference and T10 estimated data.

**Fig. 6:**
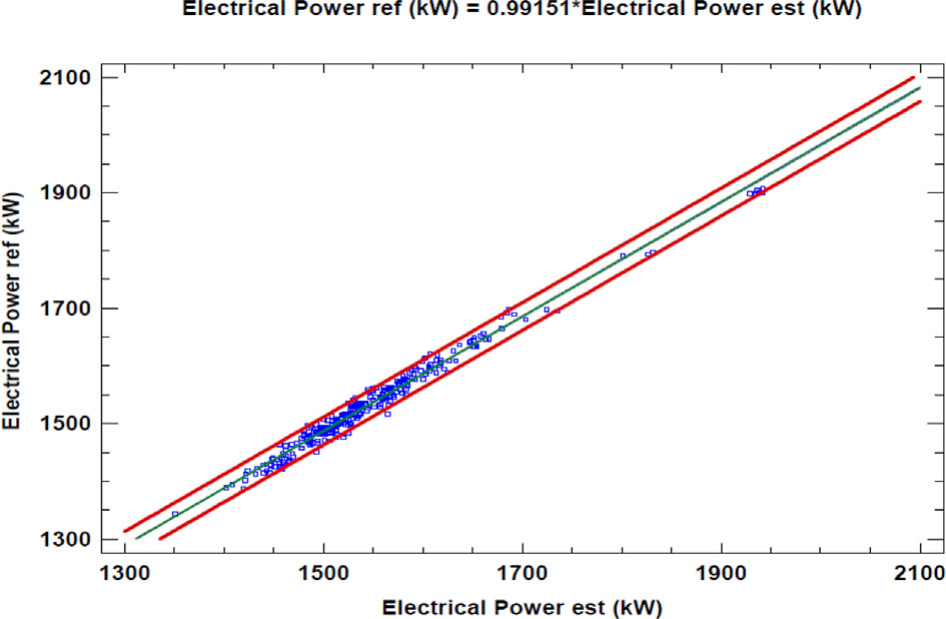
Linear regression between Electric Power reference and Electric Power estimated data.

The simulation of the model is carried out defining design characteristics, volumes of the mixing chamber, the intake and exhaust manifolds, the efficiencies of the turbine and compressor, and the efficiency of the heat exchanger among other parameters. Results were generated for a complete month of operation, in which errors no larger than 5% are obtained in variables such as the temperature at the exit of the cylinder (Temperature 8) as shown in Table 2. Also, for a better appreciation, Figs. 4, 5, and 6, shows a linear regression between the variables previously mentioned and their respective estimations. In this linear regression can be observed that the linear coefficient between the reference, which are the experimental data and the estimated values are near to one; this implies that the model has good accuracy.

**Table 2:** Measured and estimated value for some output of the model.

Variable	Measured (Mean Value)	Estimated (Mean Value)	Average absolute error [%]
Temperature 8 [°C]	614.7	613.9	0.13
Electrical Power [kW]	1659.6	1664.6	0.30
Temperature 10 [°C]	460.8	461.4	0.13

## 4 Results and discussions

**Fig. 7:**
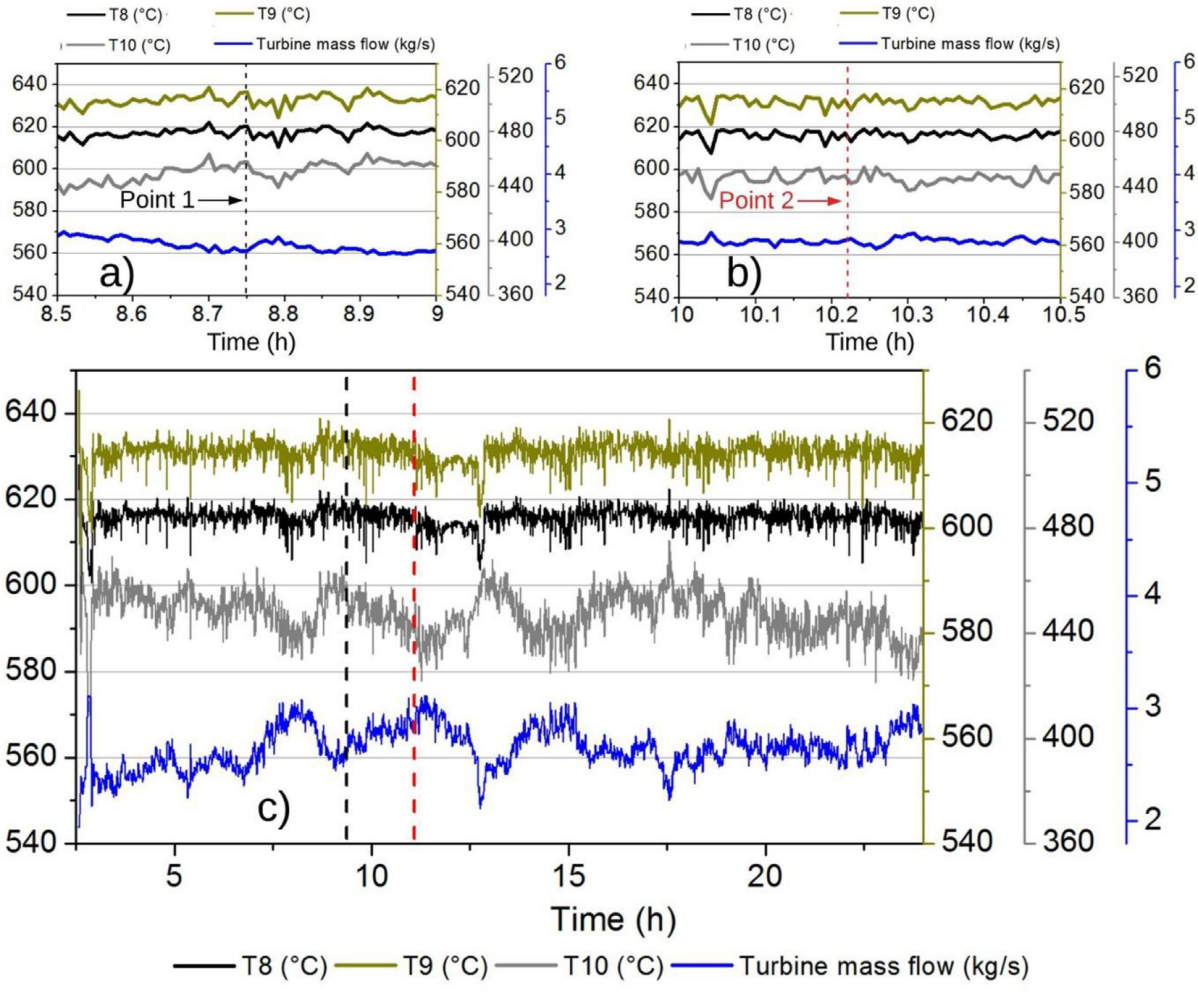
Mass Flow in the Exhaust Manifold-Turbine, Temperature 8, Temperature 9. Temperature 10 vs. Time, a) from 8.5 h to 9 h, b) from 10 h to 10.5 h and c) for 24 h.

A proportional relationship between temperatures 8, 9 and 10 is presented, which is in agreement with the theory, since, more thermal energy in the exhaust gases of the cylinder, more temperature will be obtained at the exit of the exhaust manifold and therefore at the exit of the turbocharger turbine, so a linear proportionality behavior is visible between these temperatures in Fig. 7a-c. On the other hand, the mass flow in 9, mass flow within the exhaust manifold and the Turbine, presents an inverse proportional relation concerning the temperature mentioned variables as shown comparing the engine operational values of point 1 (Fig. 7a) and point 2 (Fig. 7b). This last result could be expected due to the relationship between the gas density and its temperature when the temperature rises the density decrease, affecting proportionally the gas mass flow.

**Fig. 8:**
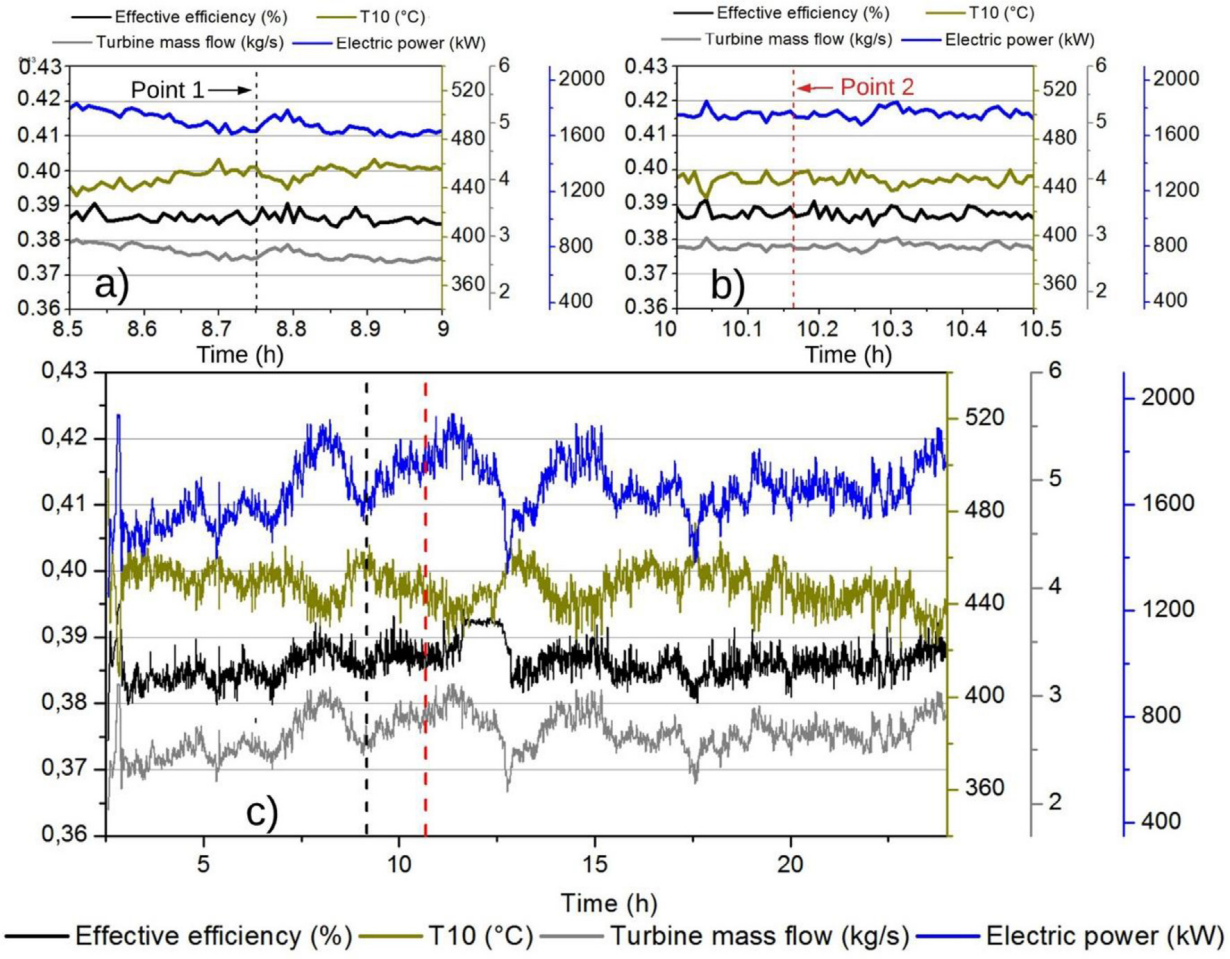
Mass Flow in the Exhaust Manifold-Turbine, Effective Efficiency, Temperature 10 and Electric Power vs. Time, a) from 8.5 h to 9 h, b) from 10 h to 10.5 h and c) for 24 h.

It is observed that the behavior of the mass flow in the outlet exhaust manifold (state 9) tends to a proportional relationship in terms of effective efficiency, as shown in Fig. 8, except in the corresponding time span between 8 and 10 h of operation as shown in Fig. 8c. The temperature at the outlet of the turbine shows a different behavior to the other parameters between 10 and 10.5 h as shown in Fgure 8b for the operatonal condition in point 2; while the temperatures at the inlet and outlet of the exhaust manifold do not vary during this period of time, the mass flow in the turbine decreases just when the values of the temperature at the outlet of the turbine increase as shown in Fgure 8a. This indicates that the mass flow entering the device ensures temperature conditions directly influencing the performance of the system. As for electrical power, in the great majority of its behavior, there is a tendency to a linear proportional relation with respect to the effective efficiency and the mass flow in (state 9). Therefore, a considerable effect of the mass flow of the turbine (state 9) and of the electrical energy generated can be seen in the effective efficiency of the engine.

**Fig. 9:**
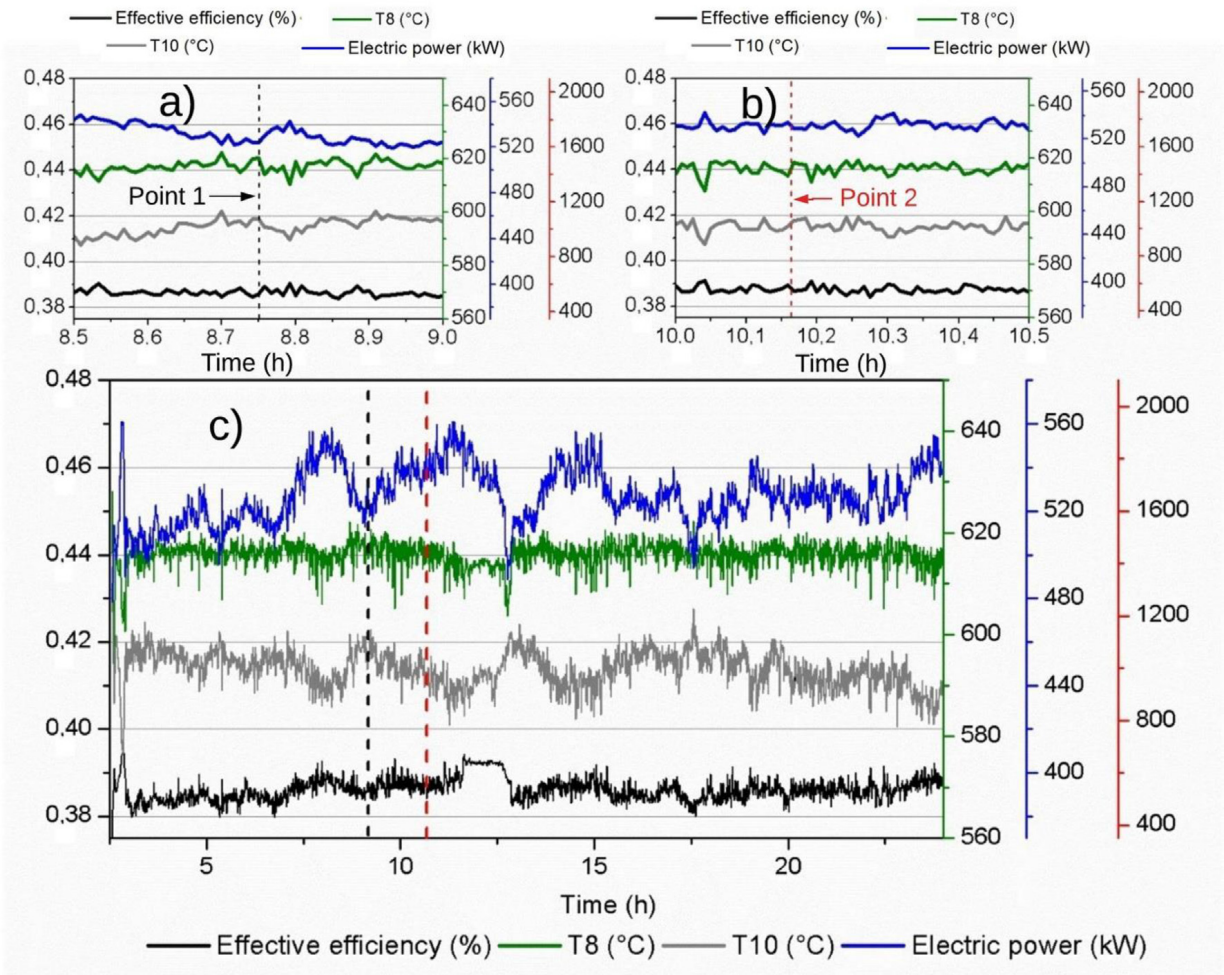
Effective Efficiency, Temperature 8, Temperature 10, and Electric Power vs Time, a) from 8.5 h to 9 h, b) from 10 h to 10.5 h and c) for 24 h.

From Fig. 9a-c, it can be found a contrary case to the one highlighted in Fig. 8, when the turbine mass flow in 9 is substituted by temperature 8. From Fig. 9c can be emphasized once again the type of previously linear relation between the electric power and effective efficiency, and about these variables compared to temperatures 8 and 10. It is possible to see the low influence of the variation of the other engine parameters on the exhaust gas temperature comparing the engine operational values of point 1 (Fig. 9a) and point 2 (Fig. 9b). The output parameter with the most stable behavior during the operating cycle is the effective efficiency, but like the other parameters, it has an anomaly between 8 and 10 working hours. The effective efficiency maintains constant values, in contrast to the power, temperature of the gases, and turbine flow, as they are linearly related.

Figs. 8 and 9 show the energy phenomena explained by the first thermodynamic law applied to the cylinder, such when the temperatures increase, the electric power and the effective decrease due to the energy losses are taken by the gas mass flow.

**Fig. 10:**
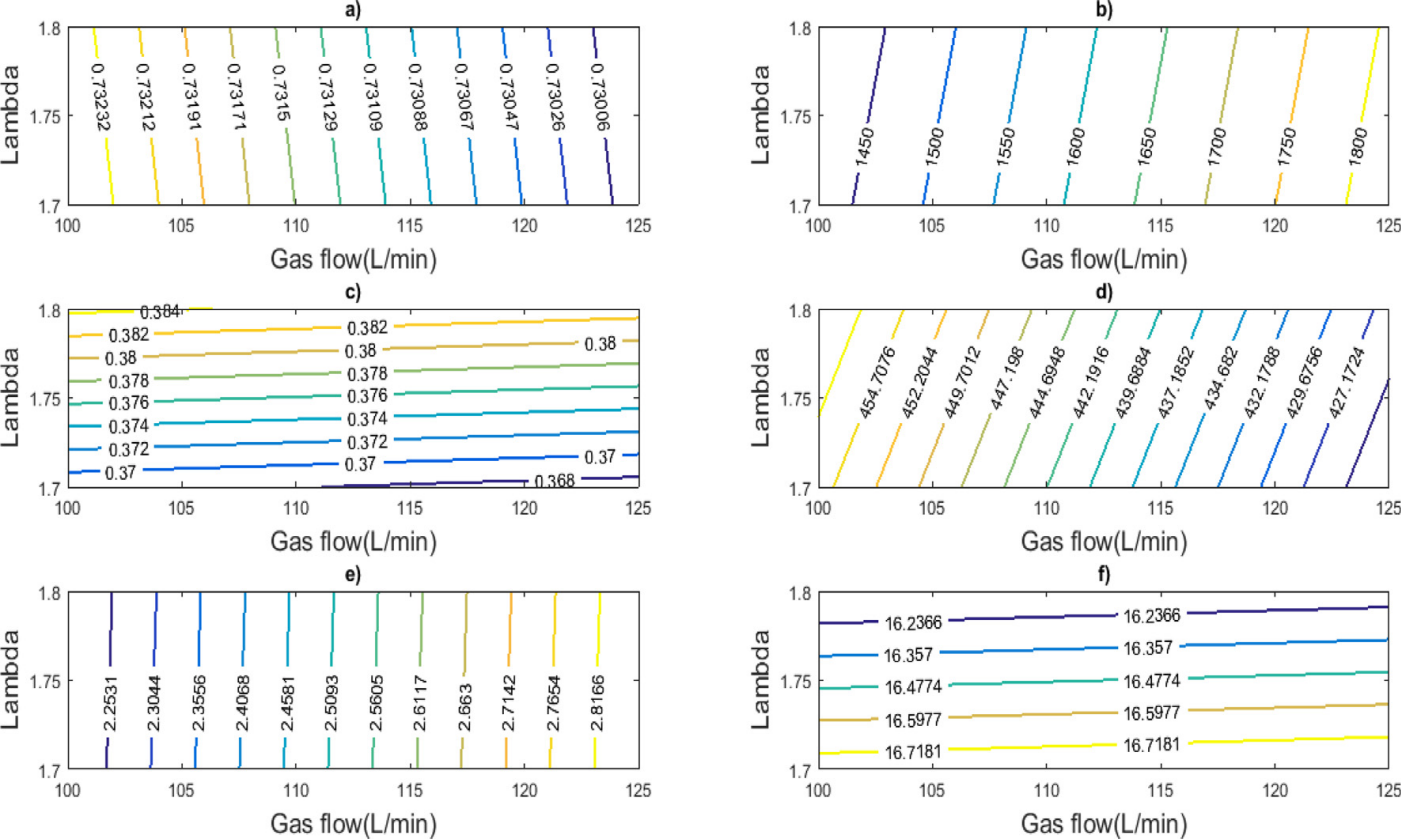
Characterization of Engine Response, a) Volumetric efficiency, b) Power output (kW), c) Effective efficiency, d) Temperature 10 (°C), e) Turbine mass flaw (kg/s), f) Specific fuel consumption (g/kWh).

From the model proposed at fixed gas pressure conditions of 1.16 bar, and a cooling water temperature of 65 °C, the average ambient temperature of 35 °C and 1500 rpm, and the operating surfaces of the Jenbacher JMS 612 GS-N. L were generated, as shown in Fig. 10, as a function of the two most relevant input variables of the engine.

These operating surfaces can be used as a tool to diagnose possible operational failures that may occur in the engine, in addition to providing information on what should be the input values of the engine to obtain a certain power output. On the other hand, this result can be used to calculate and evaluate different alternatives of waste heat recovery from the exhaust gases [33, 34]. Also, it is important to clarify that these results must be updated in case of making an adjustment or calibration of the engine combustion or use natural gas with a different stoichiometric composition from the one studied in this work.

## 5 Conclusions

As the most important contribution of this paper, the first phenomenological based semi-physical model development of a natural gas engine as a function of thermodynamic mean value represents the power generation, exhaust gas mass flow rate and temperature behavior of a widely used engine in the industrial sector, which can be used to predict and evaluate different operation condition for control strategy design, state observer development and waste heat recovery alternatives evaluation. Although the phenomenological model for chemical and industrial process has been extensively studied, the structure of the energy balance to predict the mechanical work in the cylinder used normally required the measure of the chamber pressure, which is not installed in the typical industrial gas engine to predict the heat transfer release in the four strokes of the internal combustion engine. Besides the above, the proposed model takes into account the information of typical sensor installed in this kind of gas engine to calculate the mass and energy balance in each subsystem of eth engine. This scope of the model allows expanding the application of the methodology to other gas engine implemented in the auto-generation application. Resulting this mean value model is obtained from typical measurements in this type of engine, normally the volumetric natural gas condition, lambda, and RPM, for estimating the exhaust gas thermodynamic condition, volumetric and effective efficiency of the engine with good accuracy.

Based on the phenomenological based semi-physical model developed for the cylinder-exhaust section of a natural gas 2 MW gas engine, a clear proportional relationship is denoted among all the variables studied. Of these relationships, the ones that stand out most are those between all the temperatures associated with the exhaust line with the effective efficiency and electric power generated. This makes possible to show that the efficiency in the use of the energy coming from the combustion of the natural gas used has yet to be improved, which in turn opens doors to future strategies and studies of cogeneration projects, in order to boost the current performance of the engine and its collateral consequence is the reduction of harmful emissions to the environment from the exhaust gases of the engine. The variation of lambda values affects significantly in three of the six outputs analyzed in the characterization of the engine response, which are the effective efficiency, the outlet temperature of the turbine and the specific fuel consumption.

In addition, it is proposed the use of the characterization chart of the engine oriented to identify abnormal operating conditions of the inlet and exhaust gas line, which allow obtaining a better performance of the engine during operation at standard conditions. Finally, it is recommended the development of equations and/or dynamic models to predict in real time of the exhaust gas emissions associated with the engine operating regime and thus ensure the emissions concentration desired according to international standards.

## Declarations

### Author contribution statement

Guillermo Valencia: Conceived and designed the experiments; Performed the experiments; Analyzed and interpreted the data; Contributed reagents, materials, analysis tools or data; Wrote the paper.

Jorge Duarte & Cesar Isaza: Conceived and designed the experiments; Analyzed and interpreted the data; Contributed reagents, materials, analysis tools or data.

### Funding statement

This work was supported by the Mechanical Engineering Program of Universidad del Atlántico. G. Valencia and J. Duarte were supported by The Kai Research Group.

### Competing interest statement

The authors declare no conflict of interest.

### Additional information

No additional information is available for this paper.
